# Nanocarriers for Drug Delivery: An Overview with Emphasis on Vitamin D and K Transportation

**DOI:** 10.3390/nano12081376

**Published:** 2022-04-17

**Authors:** Andreea Crintea, Alina Gabriela Dutu, Alina Sovrea, Anne-Marie Constantin, Gabriel Samasca, Aurelian Lucian Masalar, Brigitta Ifju, Eugen Linga, Lidia Neamti, Rares Andrei Tranca, Zsolt Fekete, Ciprian Nicolae Silaghi, Alexandra Marioara Craciun

**Affiliations:** 1 Department of Medical Biochemistry, Iuliu Hațieganu University of Medicine and Pharmacy, 400349 Cluj-Napoca, Romania; crintea.andreea@umfcluj.ro (A.C.); alina.dutu@umfcluj.ro (A.G.D.); masalar.aurelian@umfcluj.ro (A.L.M.); brigitta.gabriel.ifju@elearn.umfcluj.ro (B.I.); linga.eugen@umfcluj.ro (E.L.); lidya.neamti@gmail.com (L.N.); acraciun@umfcluj.ro (A.M.C.); 2 Department of Morphological Sciences, Iuliu Hațieganu University of Medicine and Pharmacy, 400349 Cluj-Napoca, Romania; simona.sovrea@umfcluj.ro (A.S.); annemarie.chindris@umfcluj.ro (A.-M.C.); 3 Department of Immunology, Iuliu Hatieganu University of Medicine and Pharmacy, 400349 Cluj-Napoca, Romania; gabriel.samasca@umfcluj.ro; 4 Department of Molecular Biology and Biotechnology, Babeș-Bolyai University, 400084 Cluj-Napoca, Romania; rrstranca@gmail.com; 5 Department of Oncology, Iuliu Hațieganu University of Medicine and Pharmacy, 400349 Cluj-Napoca, Romania; fekete.zsolt@umfcluj.ro

**Keywords:** nanocarriers, drug delivery, vitamin D, vitamin K

## Abstract

Mounting evidence shows that supplementation with vitamin D and K or their analogs induces beneficial effects in various diseases, e.g., osteoarticular, cardiovascular, or carcinogenesis. The use of drugs delivery systems via organic and inorganic nanocarriers increases the bioavailability of vitamins and analogs, enhancing their cellular delivery and effects. The nanotechnology-based dietary supplements and drugs produced by the food and pharmaceutical industries overcome the issues associated with vitamin administration, such as stability, absorption or low bioavailability. Consequently, there is a continuous interest in optimizing the carriers’ systems in order to make them more efficient and specific for the targeted tissue. In this pioneer review, we try to circumscribe the most relevant aspects related to nanocarriers for drug delivery, compare different types of nanoparticles for vitamin D and K transportation, and critically address their benefits and disadvantages.

## 1. Introduction

Nanocarriers involved in drug delivery offer several advantages when compared to conventional treatments, allowing an increase in water solubility of slightly soluble/insoluble drugs and protection against degradation and inactivation [1]. These characteristics may provide enhanced stability in comparison with traditional formulations. Further, the design of the nanocarriers involved in drug delivery facilitates the drug lingering in the bloodstream for a prolonged period, which supports more efficient accumulation at the site of action [2,3]. A necessary feature is facilitating dose reduction to patients, with a consequential decrease of the adverse effects caused by the drug itself or formulation adjuvants. By passive and active targeting, nanocarriers can accumulate at the desired action site. Scientists have currently produced nanostructured delivery systems with specific ligands bound to their surface, capable of targeting these particles to specific cells [4,5,6]. Therefore, ligands that identify the expressed or overexpressed receptors in targeted cells have been bound to nanocarriers promoting superior targeting and accumulation of the drug at the action site, thus diminishing systemic toxicity and improving its selectivity [7].

Various studies have focused their attempts on active and passive targeting of the molecular markers related to disease initiation and evolution. Considering that nanoscale drug-delivery systems provide an improved means for transporting medicines, various types of organic and inorganic nanoparticles (NPs) have been designed [6,8,9], their main objectives being the delivery of a particular molecule to its activity site and inducing enhanced pharmacological impact [10,11,12]. Apart from the targeted element, other aspects such as the nature and action of the carrier should be scrutinized while planning the drug-delivery approach [13,14,15]. The most challenging issue in drug delivery products is the system’s biocompatibility, meaning the capacity to surmount the body’s protection systems while being non-toxic and not triggering any immunological response in a organism [16,17,18]. Furthermore, other aspects of developing an adequate drug-delivery system are stability and improved interactions with the cellular membrane. Advances in comprehending biochemical interactions among tissues and drug-delivery systems have optimized nanocarriers.

To transport these new drug-delivery systems, and at the same time to enhance the efficiency of vitamins, it is necessary to continuously perfect the NPs. These systems should aim at improving the efficacy of the vitamins and patient compliance [10]. Vitamin D deficiency is associated with a higher risk of developing cardiovascular conditions, microbial infections or tumor pathogenesis [19]. Moreover, a deficiency of vitamin K can lead to bleeding, defective bone development, osteoporosis, and a greater risk of cardiovascular disease [20].

One of the ways in which vitamin D and K deficiency can be prevented would be to develop compliant therapies using nanotechnology. Therefore, the objective of this pioneer review is to highlight recent progress in the development of nanocarriers for vitamin D and K delivery and to critically compare different types of NPs for vitamin D and K transportation.

## 2. Nanocarriers for Drug Delivery: An Overview

**Figure 1:**
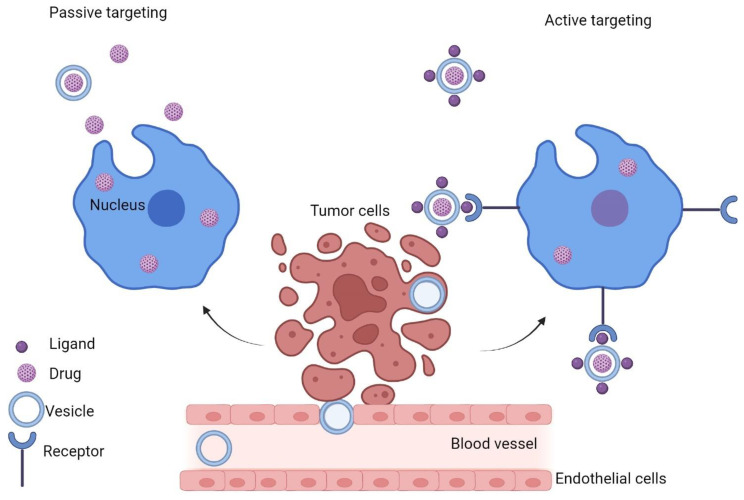
Passive and active targeting.

The most significant challenge in the medical field is to develop an antitumoral therapy [10,21,22] using passive or active targeting. In this regard, it is worth mentioning that processes like convection and diffusion directly influence the efficiency of the passive type of targeting. The phenomenon of convection occurs due to the physiological circulation of blood, being responsible for the transport of molecules of large sizes thought the orifices within the endothelium. Diffusion, on the other hand, is responsible for expelling through the membrane complexes that are either lipophilic or have a low molecular weight, in a gradient-dependent manner [23]. The enhanced permeation and retention effect may ultimately improve the assembly of nano-sized carriers. In passive targeting, the outer layer of NPs can be modified to achieve highly-specific interconnections between the transporter particle and its target when binding to receptors found in tumors [24,25,26,27]. Nevertheless, the liaison with the target cells depends on the nanocarrier’s capacity to arrive at the specific tumor location, the impact of the enhanced permeation and retention still being crucial. If considering active targeting, one should acknowledge that there will be almost no improvement in the total drug accumulation inside the tumors, with this strategy rather enhancing only cell recognition and drug uptake [24,28,29,30]. Passive and active targeting are depicted in Figure 1.

To acquire immediate curative responses of the therapeutic agents carried through nano-based delivery systems, active drug targeting approaches should be considered. This is because, in passive targeting, the NPs carry the engulfed medications passively toward the tumor cells, taking advantage of the minuscule size of the drug-delivery particles and the specific neovascularization of the tumor [1,31].

When the carrier structures disassemble, the bioactive compounds are discharged, with the degradation rate being controlled and adjusted in line with the architecture of the NPs. Polymers such as polyethylene glycol (PEG) can also be used in a coating process of nano-sized drug delivery vehicles, to improve their durability in the systemic circulation, to control their detrimental interactions with the opsonizing proteins and to practically diminish their rapid degradation and clearance [32,33,34]. Moreover, it is acknowledged that minimization of the adverse effects is a great advantage of NP usage through close modulation of the nonspecific absorption of drugs into healthy tissue [35].

There are different types of nanocarriers, generally divided into organic and inorganic, which are further presented below.

### 2.1. Organic Nanocarriers

#### 2.1.1. Nanostructured Lipid Carriers

Nanostructured lipid carriers (NLCs) were created to improve the poor drug loading and removal during storage. In the case of NLCs, the expulsion of medication triggered by the crystallization phenomenon is controlled by the lipid matrix’s remarkable structure. There are several NLC classes, such as imperfect, amorphous, or multiple NLCs. The imperfect NLCs are described by a lipid mixture composed of various fatty acids capable of inducing imperfections [36]. The imperfections in the lipid matrix create more area for drug accumulation, thus the encapsulation efficiency is increased. Amorphous NLCs are made by combining liquid with solid lipids, ultimately leading to a partition of phases which generates an oil nano-compartments occurrence in the solid lipid phase [37,38]. Entrapment of a large quantity of non-polar drugs or drug-loading improvement should be used when the drugs are not easily soluble in solid lipids and offers benefits to the solid phase of NPs that further controls the drug removal during storage [39]. Furthermore, NLCs, also known as carriers of both water-soluble and fat-soluble compounds, can robustly immobilize the contained bioactive agent. Another strategy of drug delivery nano systems reported in the literature is the manufacturing of hybrid lipid−polymer NPs that incorporate the benefits of lipid NPs and polymeric NPs [40,41].

##### Liposomes

Assembled by virtue of the self-organization of phospholipids, liposomes can be defined as artificial, small, round vesicles that are both biodegradable and biocompatible. The already well-known methods of classification of these compounds take into account their diameter, the number of bilayers, or even the manufacturing method [42]. Phospholipids are amphipathic particles having a hydrophobic extension composed of two fatty acid sequences with a number of carbon atoms ranging from 10 to 24, and a polar head which ensures their hydrophilic characteristics. The preference for phospholipids is due to their bivalent structure, since the formed bilayer can easily modify its fluidity and influence the discharge ratio of the engulfed drug [43]. Thus, liposomes are characterized by their particular structure, defined by the bilayer structure of lipids. Apart from phospholipids, cholesterol is another constituent that may be considered to obtain liposomes, since it ensures an enhanced stability of these structures [42,44]. Interacting with the carbon sequences present in liposome phospholipids, cholesterol changes their organization, consequently altering the discharge proportion of the encapsulated bioactive agent. They present increased biocompatibility like other synthetic materials and valuable drug vehicles [45,46]. Considering the above, liposomes could offer a considerable advantage as drug carriers, not only by facilitating the transport of specific medications, but also by mediating a controlled release of the therapeutic agents within a designated cell or organ [47,48]. Another benefit coming from liposomes’ usage in medicine is that they prevent the deterioration of the loaded drug, largely limiting the detrimental exposure to the environment. The drug discharge can be voluntarily activated by different techniques, such as light, ultrasound, high temperatures or even magnetism [49,50]. Further, liposomes can also change DNA, as anticancer agents, by adding characteristic molecular particles to their surface. These are the most promising tools for gene therapy, a premise proven by at least a few experiments which concluded that the suitable biodistribution of these compounds represents a great argument for choosing them as drug carriers [8]. Coating liposomes with hydrophilic polymers or sugars was shown to improve the half-life of these particles even further. The most studied models are PEG and monosialoganglioside (GM1). These overlays diminish their contact to serum proteases, hence extending their stability. As a result, liposomes’ survival is prolonged in the systemic circulation, the same strategy being used to increase the half-life of other lipid nanostructures [8,51,52].

Liposomes may become reactive if they are sensitive to extrinsic or intrinsic stimuli and exhibit modifications in their arrangement in concordance with temperature, pH, or electrostatic charge. As a reference, pH-sensitive liposomes are stable in healthy tissues. Nevertheless, tissues having a pH below 7.4 destabilize these nanospheres, leading to a discharge of the engulfed bioactive substance [53]. This is a remarkable technique that helps to intermediate the intracellular drug release, specifically when it comes to tumoral tissues. Researchers have also created functionalized liposomes, which are able to target specific molecules found on the surface of different cells, allowing their internalization into these targeted cells [54]. Consequently, there are molecules bound on liposomes’ surfaces that allow them to be guided to specific targets, ultimately increasing the quantity of therapeutic agent internalized in the neoplastic cell and limiting or preventing its internalization in healthy, normal cells. Antifungal drugs, antibiotics, and small interfering RNA (siRNA), among others, have already been considered as carriers for liposomes and used in studies as therapy alternatives for various diseases [55].

**Figure 2:**
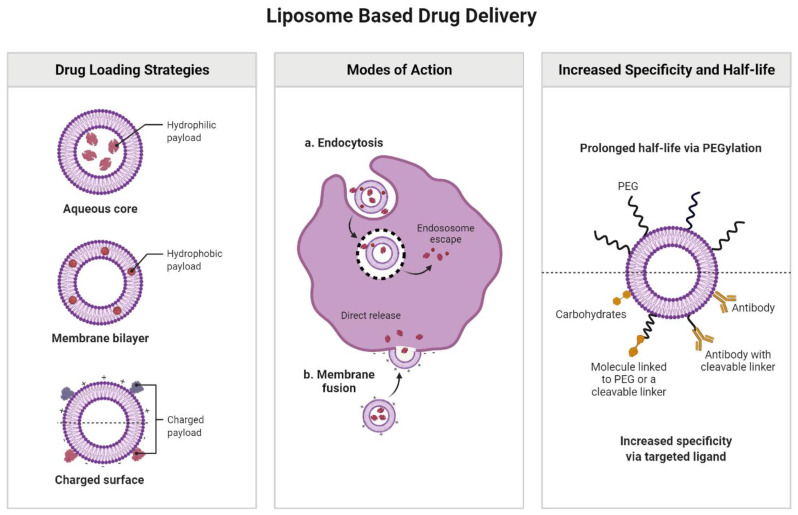
Drug loading strategies.

There are two distinct methods in which one can integrate medications into liposomes: passive and active drug packing techniques. While the passive envelopment strategy implies that the bioactive molecules are entrapped in NPs during their assembly, in case of the active loading, the therapeutic agents are packed into the intact liposomes [56]. The capacity of drug entrapment through passive approaches greatly relies on the liposome’s capability to take a specific volume of drug-containing solutions or solutes during the vesicle auto-aggregation [42,44]. In the case of water-soluble drugs, the encapsulation efficacy is directly dependent on the aqueous volume entrapped by the NPs, which, in turn, is conditioned by the liposome’s morphology, concentration of phospholipids in the dispersion, and the number of the lamella. On the other hand, when we refer to lipophilic drugs, a direct interaction with the phospholipid’s bilayer is found [57,58]. Consequently, the envelopment efficiency depends on the variety and concentration of phospholipids. In this case, morphological parameters do not influence the drug encapsulation efficacy. Conclusively, the hydrophilic drugs are loaded inside the aqueous phase of the liposome [44,58], while the hydrophobic drugs are trapped in the liposome’s bilayer (lipidic phase). The bioactive agents become captured so the lipid-soluble segment will be fixed between the constituting phospholipids of the NPs. The already manufactured unfilled liposomal vesicles for active drug entrapment are combined with the concentrated solutions of the desired drug [42,44,46,58]. After incubation, the drugs are uniformly dispersed in the liposomes via diffusion. The convenience of this method is due to the high degree of permeability for drug distribution characterizing the phospholipid’s bilayer, a fact that results in high entrapment occurring within a suitable time [59]. The drugs enter the NPs pass through the lipid bilayers, being guided solely by the concentration gradient until reaching the optimum balance between the external environment and the core of the vesicles. During dynamic loading, the hydrophilic drugs connect with the polar head groups of phospholipids, being entrapped afterwards within the liposomes [8,60]. The number of fat-soluble drugs that can permeate into a liposome is dependent on the packing constraints of the lipid bilayer [44,61]. Thus, liposome manufacturing processes for this group of drugs undergo considerable changes from one substance to another. Amphipathic medications pose difficulties in remaining inside the NPs as they can easily diffuse through the lipid bilayers. The active loading method has various conveniences, one of them being the fact that the bioactive agent is absent during the assembly process of the liposomes [42,45]. As a result, the safety precautions mandatory during the handling of toxic drugs will be diminished. The limitation of this technique is that this procedure is restricted to a variety of drugs having the properties of a weak amphiphilic base or acid which can diffuse through the bilayers exclusively in the uncharged state [62]. The different loading strategies discussed above are summarized in Figure 2.

##### Micelles

Micelles are colloidal particles, having nano-sized diameters and spherical shapes, with a polar outer surface and a non-polar interior. This type of NP may carry bioactive agents either within the hydrophobic center or bound covalently to the surface of micelles [63]. The significant benefit of the micelles consists in the fact that they can be designed and manufactured to carry fat-soluble medications very quickly. Just above their threshold concentration, micelles are built due to the self-aggregation of the amphiphiles in aqueous conditions, thus engulfing passively the fat-soluble bioactive compound partitioning into the hydrophobic medium of the micelle core [64,65,66]. If diluted below their critical concentration, the micelles break apart, releasing the drug. The features of micelles are also altered by the surrounding conditions. For example, blood contains specific compounds that can influence the potential chemical gradient created between monomeric fraction in the micelles and the surrounding aqueous phase, consequently increasing the critical micelle concentration [64,65,66]. As a result, the stable micelles in saline solution may prove to have poor stability in the blood and cause them to disperse and discharge the carried drugs in advance [67,68].

#### 2.1.2. Polymeric Nanocarriers

Polymeric NPs are versatile particles within the size range between 1 and 1000 nm, and may be given in several dosage forms. Based on their structural organization or preparation method, polymeric NPs can be classified in nano capsules or nanospheres. Polymeric NPs can be carriers of several medications for specific conditions or distinct types of treatment. The drug could be dispersed in a liquid core of oil or water, which is encapsulated by a solid polymeric membrane or could be dispersed in the polymer matrix. Among all the NPs, polymeric NPs exhibit enhanced stability and improved encapsulation effectiveness, which may be controlled by the manufacturing techniques and characteristics of the materials utilized in the formulation [50,69]. As already mentioned, a polymeric coat of the NP can provide further shelter from potential alteration and in addition delivers steric stability. Various techniques can prepare polymeric NPs using natural or synthetic polymers. The structure of these NPs may change according to the components of the formulation, developing nano capsules [69,70]. The nano capsules present a nucleus encircled by a polymeric wall, the drug being either held within the particle’s walls or adsorbed. The constituents can be functionalized and facilitate the particle’s targeting. Several polymers are used to prepare polymeric NPs [71]. Polymers must present biocompatibility, stability, suitable kinetics of biodegradation, facile processing, preservation of their characteristics for a restricted time in vivo and a slow degradation into soluble compounds [72]. Furthermore, these products must be hypotoxic after particle disassembly. Polymeric NPs can entrap a wide array of drugs after being released from the NPs according to the polymeric matrix’s solubility [69]. One of the drug release mechanisms could be via distension due to polymer hydration, followed by drug discharge through diffusion. An alternative mechanism of drug release appears through a chemical and enzymatic reaction that has as an outcome the polymer’s degradation at the release site, removing the drug from the NPs’ core [73,74]. Polymeric nanocarriers are vehicles with possible usage in multiple treatments as they can alter the diffusion of the bioactive compound, while providing excellent stability and increased absorption at the action site. They can deliver greater therapeutic efficiency and reduce the implicit harmful effects of conventional therapy [2,75].

In addition, polymeric micelles have been developed, which remain for extended periods in the systemic circulation, a property that can be conveniently used for continuous drug release. Moreover, as in the case of liposomes, polymeric micelles can be further functionalized if embossed with guiding ligand particles for targeted dispatch of drugs to designated cells and can be developed to promote drug delivery [63,76].

There are also other strategies of drug delivery nano systems reported in the literature such as the manufacturing of hybrid lipid−polymer NPs that incorporate the benefits of both lipid and polymeric NPs [40,41].

#### 2.1.3. Nano-Emulsion Technology

Nano-emulsion can be considered a protocol which uses small particles that vary from 50 to 500 nm. By using the nano-emulsion technology, semitransparent and low viscosity solutions can be obtained [77]. The main compounds used in the manufacturing process are oil, water, and a surfactant or co-surfactant. The assembly procedure uses surfactants that have already been authorized for human use, while also demanding a reduced concentration of surfactants in contrast to micro-emulsion technology. Furthermore, these solutions may also be used in lamellar phases, forming thin liquid coats around the nano-emulsion droplets, which may result in exclusive drug delivery characteristics [78,79]. Nano-emulsion technology has been advantageous in developing different delivery products, which include creams, liquid solutions, and foams. The procedure is also largely utilized in solvents for lipophilic drugs, covering their unpleasant taste [80,81,82], but improving bioavailability and absorption, while also suppressing irregularities in absorption. Consequently, the drugs may be administered through multiple routes, therefore being utilized to achieve immediate or late curative effects of the treatment [83,84]. It was also proven that oil-soluble active substances used in nano-emulsion technology may lead to enhanced cellular uptake of the bioactive content and protect the presumably unstable drug molecules from harmful factors, such as light and enzymatic or oxidative degradation. Through the nano-emulsion assembly protocols, controlled drugs with various chemical and physical properties can be delivered to specific cells or tissues [21,85]. Consequently, the final delivery system can solubilize the lipophilic drug and increase bioavailability when the drug is orally administered [21].

### 2.2. Inorganic Nanocarriers

Inorganic elements, such as gold, are frequently considered as raw materials in the production of metallic NPs. Metallic NPs can be largely characterized as greatly organized structures, in a three-dimensional manner [86]. Being more malleable than other types of NPs, these nano-sized particles have the possibility of regulating their shape, size, design, composition, or encapsulation. Even if inorganic NPs show several benefits, a series of weaknesses should be still considered with respect to biomedical applications. The most important limitations of these inorganic NPs are the lack of ability to load drugs into their configuration and the relatively well-known cytotoxicity and blood-related adverse effects, facts that depend on the concentration of the carried agent, their size, and exposure time [4,87,88]. The synergic combination of bioactive molecules and inorganic materials in nanocarriers intends to overcome traditional challenges posed by the pharmaceutical domain, as some classically-administered drugs exhibit low solubility and specificity, poor bioavailability, and low treatment efficacy [2,21,89].

Gold nanostructures have been used due to their remarkable optical properties. Beyond their optical properties, gold nanoparticles (GNPs) exhibit low reactivity and toxicity, biocompatibility, and high capacity of functionalization, becoming great candidate materials for biomedical applications [90,91]. Currently, diverse strategies for gold nanoparticle synthesis with specific architectures have been reported, spherical nanoparticles and nanorods being the most used gold nano-sized structures in the biomedical field [92,93]. In addition, they may be great candidates for in vivo experiments. A modified method is to coat nanoparticles with PEG, a polymer that enhances stability and prevents opsonization in biological environments [94,95,96]. The combination of molecules and polymers on the GNPs’ surface is an excellent approach to amplify their half-life in the bloodstream and, ultimately, to promote their biodistribution and tissue-targeting capacities. GNPs exhibit convenient water solubility, excellent photostability, and a high surface-to-volume ratio, increasing surface functionalization ability [97,98]. Various cargo molecules can be encased within this type of nanocarrier, varying from anticancer drugs to proteins and nucleic acids. In addition, they can be conjugated with a large array of materials, linked through covalent bounds via thiol/amine groups, producing functionalized gold nanoconjugates [99,100,101]. Moreover, their bioinert properties and the versatility of surface modification make GNPs strong candidates for drug delivery purposes. Certainly, a good trigger for the controlled release of the engulfed bioagents would be their photothermal conversion [102,103,104]. Drug-delivery systems, as already known, rely on the entrapment of a drug inside a carrier or on the conjugation of drugs on the surface of these carrier particles. Thus, therapeutic agents may enhance their selectivity, pharmacokinetics, and effectiveness overall, while having fewer side effects [1,16,21,105]. By targeting therapeutic agents in functionalized GNPs, a synergic effect may be further obtained. Despite all the progress made in recent decades regarding the surface modification and synthesis procedures of gold-based NPs, their biological stability for in vivo factual utilization in the clinical field remains challenging [106,107,108].

Other promising inorganic nanoparticles for biomedical applications are silica NPs. Currently, the literature describes two types of silica NPs: mesoporous and solid. Both are promising drug delivery systems: the surface can be functionalized to interact specifical with cells or receptors, whereas the internal structure can be modified to assure the control of releasing the drug payload. Besides drug delivery systems, other applications of silica NPs include: gene delivery, protein delivery, bar-coding tags, DNA detection, imaging, and diagnosis [109].

Carbon NPs can vary in form, ranging between different carbon-based NPs and nanocomposites: carbon nanotubes, carbon nanofibers, grapheme, and fullerenes. Due to their capacity of being functionalized, they can be used in medical or industrial applications. Although showing advantageous characteristics (tensile strength, specific surface area, electrical conductivity), carbon NPs present certain weaknesses: they are insoluble in aqueous or organic solvents and tendency to form agglomerates. These limitations can be rectified by functionalization [110].

In biomedical applications, magnetic NPs have an essential role because of their exclusive magnetic properties. Particularly important are iron oxide nanoparticles, due to their properties of manipulating particle motion, useful in imaging external magnetic fields. Other important properties include high biocompatibility, low cytotoxicity, and high drug loading capacity. Iron oxide nanoparticles are widely used in magnetically triggered drug release, magnetic particle imaging, and cancer therapies [89,111].

As a conclusion, Table 1 summarizes the characteristics, strengths and weaknesses, as well as data on the clinical use of all the organic and inorganic NPs discussed above.

## 3. Nanocarriers for Delivery of Vitamin D and K

### 3.1. Vitamin D Metabolism: A Brief Overview

The fat-soluble vitamin D is associated with the metabolism of calcium and phosphorus by facilitating the absorption of calcium from the intestine and contributing to bone mineralization. Vitamin Ds are a group of sterols that have a hormone-like action [119,120,121]. The active molecule 1,25-dihydroxycholecalciferol binds to nuclear receptor proteins. The dietary vitamin D compounds (D2 and D3) are absorbed into the enterocytes of jejunum and ileum, from where the nascent chylomicrons will carry vitamin D into the bloodstream [122]. After being synthesized in the epidermis, the endogenous vitamin D3 is attached to the vitamin D-binding protein present in the plasma and transferred to the liver for further processing. The processing pathways of vitamin D2 and D3 are alike. Briefly, vitamin D is hydroxylated to 25-hydroxyl vitamin D (25-OHD) by hepatic 25-hydroxylase in the endoplasmic reticulum compartment of the hepatocyte [123,124,125]. The 25-OHD synthesized in the liver further undergoes hydroxylation, the responsible enzyme being 1-alpha-hydroxylase, and 1,25-dihydroxy vitamin D is produced, which is the active type of vitamin D. This bioactive form of vitamin D stimulates the intestinal absorption of important minerals, such as calcium and phosphorus [123,126,127]. Vitamin D and its analogs have been shown to induce apoptosis of tumor cells or promote their differentiation into a mature and less-aggressive form of the cell lines. Although there are mounting studies on this topic, the evidence is conflicting in terms of the beneficial effects of vitamin D in several malignancies [125,126,128,129].

### 3.2. Synopsis on Vitamin K Metabolism

Vitamin K is an essential fat-soluble vitamin whose crucial role is in post-translational modification of a set of proteins referred to as vitamin K-dependent proteins, or Gla proteins (due to the presence of gamma carboxyglutamic residues), which are engaged in several physiological and pathological mechanisms in the human body such as coagulation, vascular calcification, and bone metabolism [130]. There are several known isoforms of vitamin K: phylloquinone or vitamin K1 (found in green leafy vegetables), menaquinones or vitamin K2 (found in fermented cheese and soybeans), and the synthetic compounds menadione or vitamin K3. The absorption of vitamin K depends on its integration into mixed micelles in the intestinal lumen, demanding the presence of both bile acids and products of pancreatic enzymes [131,132]. Dietary vitamin K is absorbed in the proximal small intestine by active transport and is integrated into chylomicrons, which are then secreted into the lymph and enter the bloodstream. Chylomicrons and very-low-density lipoproteins transport phylloquinone through the bloodstream, which is then taken up by the extrahepatic tissues to synthesize menaquinone, the main vitamin K deposit form found outside the liver [133]. In the terminal ileum area where bile salts are present, menaquinone is absorbed. It is worth mentioning that menaquinones developed by the colonic bacteria are poorly absorbed, since they remain firmly bound to the membranes of the bacterial cells due to the lack of bile salts [134,135]. On the other hand, menadione is rapidly metabolized so that only a small ratio is transformed to menaquinone-4, the bioactive form [136].

### 3.3. Vitamin D and K Awareness

Dedicated research conducted by the Centers for Disease Control and Prevention (CDC) and other institutes has shown that the deficiencies of critical vitamins seem to be even more dramatic than we may believe [137]. Specifically, a study organized by the CDC between 2015 and 2016, and another investigation carried out from 1999 to 2014, concluded that vitamin D supplements taken by the US population are greatly unbalanced [138,139,140]. With more than 18% of the population taking 1000 IU or more of vitamin D daily (considered as a high dose) between 2013 and 2014 and with only a total of 28% of all individuals taking vitamin D supplements in the next year interval, it was clear that the US population is not properly informed on the appropriate administration of vitamin D [141,142,143,144].

A study on a large population carried out in 2019 by Lips et al. [145], showed similar unfortunate results with regard to serum concentration of vitamin D in Europe and even worse tendencies in the Middle East countries. Briefly, while in Europe 20–60% of the population suffers from vitamin D hypovitaminosis, in the Middle East the demographic share of this deficiency may reach 80%. In the case of vitamin K, mainly due to dietary habits, the common European generally has higher intakes than the superior recommended limits widely agreed [146].

Due to poor dietary perspectives and inadequate medical advice, African and Asian countries have the worst prognoses for vitamin D and/or K hypovitaminosis. This has been scientifically proven repeatedly, culminating with data showing a high prevalence of Vitamin K deficiency bleeding (VKDB) among Asian countries and rickets in African populations (which occurs especially in infants) [133,146,147,148]. As regards Australia and New Zeeland, the inhabitants mainly take their necessary dose of vitamin D through sun exposure, and only a few groups of people are at risk of hypovitaminosis due to job-related activities. Concerning vitamin K levels, both countries have relatively clear governmental policies that encourage the administration of vitamin K to neonates, in order to prevent VDKB development. The same governmental support is also given in case of vitamin D supplements intake for those found at risk of hypovitaminosis [149,150,151].

All things considered, even if developed countries benefit from an appropriate dietary intake, superior medical care, and protective governmental measures, hypovitaminosis conditions may still pose concerns, while in the underdeveloped nations, these deficits have serious implications, as presented above. However, although both compounds can be acquired from food, beverages, or synthesized endogenously, it is safe to assume that the current levels of vitamins D and K globally predict some rather threatening perspectives, which may be greatly alleviated through the use of nanocarriers in dietary supplements.

### 3.4. Delivery of Vitamin D and K Using Nanocarriers

With more than 409 clinical trials and more than 50 approved formulations by the Food and Drug Administration (FDA) involving nanomedicine concepts in the past 12 years, the development of nano-based therapies can still be considered a highly emergent field [152,153]. Even if this field may have been overpromoted, it is our strong belief that with revenues, nanobiotechnology will finally thrive and dramatically improve the quality of life worldwide.

Until now, nanocarriers have been mainly designed to fight infections, cancers, rare diseases, or even serve as bone substitutes. With the polymer-based formulations being the most frequent choice for the already approved drugs, these NPs can have different matrices, from nanocrystals to liposomes or inorganic complexes [21,154,155]. The most common benefits brought by NPs in the already approved formulations are reported to be decreased (systemic) toxicity and increased delivery to the disease site, implicitly, greater stability of the loaded agent (mainly proteins), and prolonged release. These benefits, as expected, also overlap with the main reasons for which nanocarriers were considered as drug carriers in the first place [156,157]. We emphasize this information, even if it seems redundant, as it may help to put in proper context the current chapter.

It is worth mentioning that even if nano-sized carriers were mainly developed for rather complex health issues, like those mentioned above, they may also find applicability in enhancing the delivery of vitamins [158,159,160]. Considered as an important subtype of supplements, these compounds are frequently chosen as efficient prophylactic agents, being ubiquitously available, generally procured with no need of a specialized medical prescription [161]. However, since vitamin deficiency can lead to serious health concerns, such as anemia, vision or memory loss, fractures, or increased risk of several types of cancer, this medication segment cannot be neglected.

Falling under the category of lipophilic vitamins, both vitamin D and vitamin K may present poor bioavailability, due to the enzymes and an improper chemical environment found in the gastrointestinal tract [162]. As the main way of supplements delivery is via oral administration, the usable intake quantities may be much lower than expected due to absorption, solubility, and molecular stability concerns [163,164]. In addition, the absorption of these two nutrients may further interfere with host diseases (especially liver or gut disorders), diet, the age of the host, or the usage of anti-obesity drugs. In addition, it was proven that vitamin D and K share the same pathways for their uptake, so they may be considered competitors with regard to their intestinal absorption [165]. Taking all the possible aspects into account, the importance of developing novel delivery strategies using nano-dimensioned shells to protect these molecules and improve their systemic assimilation remains conclusive. Thus, we summarize the NP-based formulations appropriate for the encapsulation of vitamins D and K in Table 2 and Table 3.

As we note in Table 1, in an expected manner, organic NPs are largely preferred for vitamin D delivery to inorganic ones, primarily due to the safety concerns raised by the second category.

Still, besides the examples mentioned in Table 1, it is worth mentioning that inorganic NPs made of calcium phosphate, or from copper oxide or selenium, were successfully used to regulate the vitamin levels in serum of rheumatoid patients or in women with infertility, respectively [180].

Thus, nano-sized systems may also be used as tools that act rather indirectly in therapies, and punctually in health conditions that may be affected by detrimental vitamin D levels.

**Table 3:** Nanoparticle-based formulations for the encapsulation of vitamin K.

Encapsulated Vitamin	Nanocarrier Type	Findings	References
Vitamin K	Nanostructured lipid carriers (NLCs)	NLCs may not be the best nanocarriers to transport vitamin K1, since the encapsulation yield of the bioactive substance seems to be poor (<5%).	[181]
		Due to its hydrophobic character, similar to vitamin D, vitamin K tends to incorporate into the lipidic matrix of the NPs, which not only reduces the concentration of the load but may also pose issues related to the NP stability and size.	[182]
	Micelles	Vitamin K encapsulated in micelles made of EPC, DSPE-PEG 2000 and glycocholic acid showed enhanced stability in an acidic environment (mimicking gastric fluid) when compared to other traditional orally administered vitamin K supplements.	[183]
		The PEG coating was highly recommended for micelles containing this compound, in order to avoid the coalescence of vitamin K-containing NPs due to the low intragastric pH.	[184]
		The ability to overcome malabsorption of vitamin K under cholestatic conditions by using micelles loaded with this nutrient was considered in at least two independent studies.	[185]
	Liposomes	Liposome−vitamin K formulations were designed as an aerosol for topical delivery, expected to overcome acneiform reactions, that may also prevent the formation of wrinkles.	[186]
		The simil-microfluidic method is regarded as a highly efficient procedure to obtain stable and highly loaded NPs that encapsulate vitamin D3, K2, E, and other compounds (such as curcumin extract).	[187]
		The entrapment efficiency of vitamin K2 into nanoliposomes is greater than the encapsulation of vitamin D3.	[188]
	Inorganic NPs	Biomimetic hydroxyapatite/poly xylitol sebacic adibate/vitamin K nanocomposites proved to have the appropriate roughness to adhere to natural damaged bone and enhance its regeneration through vitamin K activity.	[189]

As for the alternatives available for vitamin K encapsulation, we note there are only a few relevant studies. Surprisingly, there are no pertinent investigations on the inorganic NPs that could be used for vitamin K delivery. The slightly reduced interest in vitamin K transportation via nano-sized shells, in comparison to vitamin D nanocarriers, could be explained either by the less frequent occurrence of vitamin K deficiency in the population or through less investigation related to it.

Many of the formulations mentioned in Table 1 and Table 2 have a generous range of uses, from beverage or food fortification with vitamins to the production of cosmetic products, anti-tumoral medications, and bone regeneration. There are also commercially available liposomal formulations that encapsulate both vitamins D3 and K2.

Even if each nanocarrier type presents both advantages and disadvantages under certain conditions, in perspective we are looking forward to comparative experimental studies to outline the benefits and drawbacks of NPs with different matrices. As the acknowledgment of the need for a healthier lifestyle and the importance of micronutrients is rising, even due to the pandemic situation, we expect that comparative studies would be further considered. Nevertheless, this is a pioneer review on nanocarriers that have proven to be suitable for vitamin D and K transport to tissular level.

An interesting, yet niche topic, regarding the evolution and characteristics of various types of NPs is represented by lipid NP formulations. More than three decades ago, when they were first produced, these nanocarriers presented several advantages over other conventional carriers (like liposomes), including but not limited to improved site-specificity of drug delivery or a precisely controlled release of the loaded compounds [181,190,191]. The first generation of these lipid NPs, known as solid lipid nanoparticles (SLNs), was developed in parallel in 1991 by different specialists in Italy and Germany [163,192,193,194,195]. With regard to vitamin delivery through SLNs, although studies are showing a good loading capacity of the vitamin D2 and vitamin K1 in such nanostructures and relatively good storage stability of these NPs, the general tendency is to consider these nanocarriers as rather primitive, unstable and inefficient structures [196,197]. This is the reason why we decided to rather exclude these formulations from our tables, but still mention them to provide an evolutionary view of the lipid-matrix-based NPs in the context of possible nano-sized systems used for vitamin delivery [198,199]. Consisting of a blend of solid and liquid lipids, NLCs came to solve mainly the poor loading capacities of SLNs and the long-storage issues related to these first-generation NPs [34,40]. According to the literature, there are still concerns related to the usage of these NLCs as nanocarriers, although the improvements over SLNs cannot be denied, a fact reflected as well in Table 1 and Table 2. The dramatic improvement brought by these novel structures comes from their very structure, which is much more flexible, allowing a greater quantity of drugs/vitamins to be loaded, while also preventing the lipid crystallization phenomenon that would ultimately lead to the expulsion of the bioactive compound, a process that occurs frequently in the case of SLNs [200,201,202]. Still, both types of NPs share a common incapacity: the poor integration of hydrophilic drugs. For this purpose, lipid drug conjugates were developed in 2001 in order to facilitate the delivery of bio-compounds having any polarity [203].

**Figure 3:**
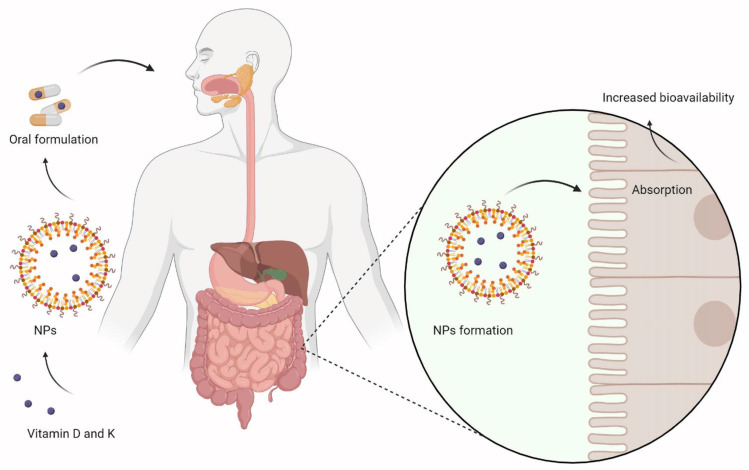
Use of nanocarriers to improve oral delivery of vitamins D and K.

In Figure 3 we envisage the use of oral formulations with NPs for vitamin D and K delivery. Oral administration of NPs delivering vitamin D and K is an efficient route of administration, as other delivery methods are associated with various issues: adipose tissue accumulation or decreased biocompatibility [204]. Depending on the delivery system used, digestion of the NPs and release of the vitamins can be made by different mechanisms. Protein digestive enzymes in the stomach can release the vitamins from the protein-based delivery systems, decreasing their bioavailability. If the NPs are lipid-based, they are hydrolyzed by lipases directly in the intestinal lumen, exposing the vitamins to the enterocytes, increasing the intestinal passive absorption and the their bioavailability [205].

Another perspective that was not encountered in the consulted studies, but may be very effective as a clinical approach, would be a hybrid strategy for the gavage of the two vitamins in parallel [161,162]. Specifically, because these two lipophilic compounds may be competitors when it comes to their intestinal absorption, taking a conventional orally-given capsule could provide a fast systemic release of vitamin D while a nanocarrier with prolonged release of vitamin K would serve as a mediator that would prevent the two vitamins from being absorbed in the same time [206]. Even if we could not find any mention of such strategy, this approach should be at least considered for further in vivo studies, if not tried to be applied as a clinical plan.

There are also alternative nanocarriers not included in our tables, since they rather represent isolated efforts to discover emerging formulations, which are not yet validated or widely used. An example in this respect, consisting of NPs made from alginate derivatives, was proved to be not only resistant to a simulated gastric fluid, but also partly immune to an experimental intestinal juice, a fact that would ultimately lead to a sustained, prolonged absorption of a high dose of vitamin D3 [207].

Recently, vitamin D-loaded nano emulsions were also considered in several studies as a viable solution for its delivery, with promising results in humans and rats [208]. Besides the expected advantages, such as improved systemic intake for patients suffering from malabsorption diseases and offering a great candidate for food fortification, remarkably these vitamin D-engulfing nano emulsions were proven to mediate hepatoprotective activity in rats with liver injury caused by a high-fat diet [166,209]. Corroborating with the usual hypovitaminosis D observed in coexistence with the non-alcoholic fatty liver disease (NAFLD), nano-emulsions based on fish oil may dramatically improve the prognosis of NAFLD patients [210,211,212]. This is because such kind of fat was already shown to improve vitamin D bio accessibility in the first place when compared to this nutrient found in the free form, and secondly, due to the great quantity of ω−3 fatty acids found in fish oil, compounds already largely known for their hepatoprotective effects, especially when we refer to NAFLD prevention or alleviation [213,214].

Nano-emulsion formulations showed promising results also regarding the delivery of vitamin K. Even more interestingly, in the case of vitamin K1, scientists successfully developed an aqueous solution containing the bioactive element, which could be sprayed on the skin and absorbed into it [215]. Having also enhanced stability in different storage conditions, this experiment could provide a factual, attractive variant for the materialization of commercial products that facilitate topical administration of vitamin K1 [186,216,217]. Furthermore, as in vitamin D nano emulsions, this kind of formulation can have collateral benefits, other than providing the necessary quantities of supplements in hypovitaminosis cases. In this respect, nano-based emulsions containing vitamin K2, conjugated with a sialic acid (SA)–cholesterol complex, exhibited clear and specific anti-tumoral activity in a murine sarcoma cell line, due to the known antineoplastic activity of this vitamin and the high affinity of the SA-cholesterol conjugate for tumoral markers [218].

All the above examples clearly outline the versatility of the nano-sized formulations involving each of these two families of compounds (vitamin D and/or vitamin K), inviting intense research on the matter, while also highlighting the opportunities that may emerge from these studies.

Despite still exhibiting safety issues, inorganic NPs may still create favorable circumstances for the delivery of vitamins. Even though this segment was poorly represented in Table 1 and Table 2, it is worth briefly mentioning some of the most promising experiments performed on nano-sized inorganic particles. For example, by conjugating vitamin D with GNPs, Shi et al. [219] proposed a system that not only inhibits the osteoclast differentiation process but also dramatically decreases the quantity of the local reactive oxygen species in cases of osteoporosis [219,220,221]. Due to these antioxidant properties, we might also assume that this technology could be largely tested and applied as a therapeutic agent in other oxidative stress-induced conditions, such as cancers, diabetes, Alzheimer’s disease, Parkinson’s disease, or others [222]. Vitamin D-conjugated GNPs can also play a vital role in nano-based bone tissue engineering, which was partially confirmed by Nah et al. [223], since great enhancements have been achieved in this domain lately, with GNPs being recognized as important actors in the field. In favor of this hypothesis, we also want to evoke an experiment that used vitamin D-loaded hydroxyapatite nanocomposites [224]. Ingeniously built, these nanocomposites can provide not merely the necessary vitamin levels for sustained bone mineralization, but also inorganic agents, such as Ca^2+^ and PO_4_^3−^, contained in the matrix of the NPs, that is crucial for bone tissue regeneration [225].

Unfortunately, as already mentioned, we could not find any relevant examples of nano-based formulations for vitamin K delivery that are based on inorganic materials. This fact strengthens the premise that this niched approach of delivery for these specific classes of compounds is still largely unexplored, presumably posing rich opportunities. However, it is our conviction that similar strategies used in the case of vitamin D can also be considered for the development of inorganic NPs carrying vitamin K. This assumption is mainly based on the similar polarity of these two vitamins, as well as on the already-existing commercial formulations that encapsulate both these vitamins under the same conditions and in a single common process. Moreover, the actual manufacturing strategies used to obtain organic NPs are largely similar in the cases of both vitamins D and K. However, even if one could rely on the existing experiments that describe the synthesis of inorganic nano systems used for vitamin D delivery, notable struggles may occur when trying to obtain conjugates between vitamin K and mineral substrates through the same techniques used for vitamin D composites. This is because, even if these two classes of compounds are both fat-soluble, sharing some physiological functions, and largely found in synergistic processes, their chemical structures present many differences, which may lead to incompatible associations with very same inorganic conjugates.

Considering all the above, it is safe to assume that there are still many opportunities in this field, from both perspectives of academic research and industry innovation. As already stated, we believe that these nano-based solutions could, at least partially, solve the factual issue of vitamin D and K deficiency or hypovitaminosis worldwide. Even if we consider bioactive-containing nano emulsions incorporated in common topical creams, and loaded micelles that overcome malabsorption or GNPs as a solution for bone tissue engineering, it is clear that these innovative products hold some answers for current global vitamin intake issues. Therefore, further investment and research on this field can lead to productive outcomes.

## 4. Conclusions

With the growing understanding of vitamins’ health effects and the harmful risks associated with vitamin D and K deficiency, discovering new solutions has become critical within the scientific community. Consequently, nanotechnology has emerged as a suitable answer via the capacity to engineer carriers at nanoscale level which presents numerous beneficial characteristics encapsulated into one multifunctional NP.

This is a pioneer review on nanocarriers for vitamin D and K, which could be helpful to optimize the already-described systems and make them more efficient and specific to a target tissue. Nanocarriers for drug delivery represent a novel medical entity, thus further techniques are required to facilitate the translation of NP-based medications from discovery to development, accelerating the transformation of nanotechnology-proven advantages into real solutions for patients.

## Figures and Tables

**Table 1:** A brief overview of organic and inorganic nanocarriers.

Type of Particles	Strengths	Weaknesses	Features	Biocompatibility	Clinical Uses	References
Organic nanocarriers						
Liposomes	Facilitate the transport of specific medications; Prevent deterioration of the loaded drug; Increased half-life when coated; High permeability of drug distribution.	Sensitive to extrinsic and intrinsic stimuli; Limited variety of drugs that can be delivered.	Small, round vesicles; Amphipathic particles; Bilayer structure of phospholipids and cholesterol; Functionalization with PEG.	High	Controlled release of therapeutic agents in a specific tissue or organ.	[8,42,44,45,46,47,48,49,50,51,52,53,54,55,56,57,58,60,61,62]
Micelles	Prolonged, continuous drug release; Carry fat-soluble medications very quickly.	Poor stability in blood; Use only for lipophilic drugs.	Colloidal particles; Nano-sized diameters; Spherical shapes; Polar outer surface; Non-polar interior.	High	Drug-delivery systems.	[64,65,66,112]
Polymeric	Versatile; Enhanced stability; Improved encapsulation efficacy; Steric stability; Great therapeutic efficiency.	Poor drug encapsulation for certain hydrophilic drugs; Possible drug leakage; Toxic degradation, monomer aggregation.	May differ in concentration and composition, in size, shape, surface characteristics, dispersion state.	Depends on the polymer used	Ocular drug delivery; Cancer diagnosis; Oncologic treatment; Stimuli-responsive and triggered release systems.	[42,43,44,45,46,47,48,49,113]
Inorganic nanocarriers						
Gold	Low reactivity and toxicity; High capacity of functionalization; Soluble in water; High surface-to-volume ratio and photostability; Bioinert; Carry different types of molecules.	Decreased biological stability in vivo.	Different shapes: spherical, rods; Functionalized with PEG.	High	Biomedical applications—Genomics, Immunoassays, etc. Photothermal therapy; Drug carriers.	[93,94,95,96,98,99,100,101,102,103,104,105,106,107,108,109,110,111,114]
Silica	Versatility; High surface area; Homogenous distribution; Non-toxicity; Flexible; High drug load capacity; Easy functionalization.	Surface density of silanol groups; Metabolic changes.	Functionalized with PEG.	High	Biological imaging; Delivery of drugs, genetic material, proteins. DNA barcoding	[115,116]
Carbon	Tensile strength; Electrical conductivity; Specific surface area.	Tendency to agglomerate; Insoluble in aqueous and organic solvents.	Different properties depending on shape, interactions between carbons; Functionalized with PEG.	Graphene NPs are biocompatible.	Biomedical; Industrial.	[117]
Iron Oxide	Low cytotoxicity; Magnetic properties; Low price.	Conjugation with DNA, proteins, organic dyes	Spherical or irregular shape.	High	Cancer therapies; Imaging and diagnosis; Magnetically triggered drug release.	[92,118]

**Table 2:** Nanoparticle-based formulations for the encapsulation of vitamin D.

Encapsulated Vitamin	Nanocarrier Type	Findings	References
Vitamin D	Nanostructured lipid carriers (NLCs)	D3-NLC formulations determined faster systemic absorption and prolonged presence of the bioactive compound in plasma.	[166,167]
		Poloxamer407 was proven as the best non-ionic surfactant to stabilize D3-containing NPs.	[168]
		Combined with doxorubicin, vitamin D-loaded NLCs can enhance the efficacy of chemotherapy in breast cancer.	[169]
	Micelles	Casein micelles protect the encapsulated vitamin D2 against UV-light induced deterioration.	[170]
		Vitamin D2 presents a great affinity for caseins (which may auto-assemble in micelles).	[170]
		Chitosan use in micelles formulations may diminish vitamin D bioavailability by up to 37%.	[171]
	Liposomes	Liposome-D3 loaded nano capsules were successfully used as anti-photoaging agents when applied directly on the skin	[172]
		NPs membrane stability was reported to be affected by vitamin D3; the issue was amended by chitosan coating.	[173]
	Polymers	Tyro sphere formulations carrying vitamin D3, already used to entrap other drugs, such as paclitaxel, exhibited a much greater skin permeation for topical administration.	[174]
		The encapsulation of vitamin D in the so-called BMC polymer protected the bioactive compound even after the remarkable challenge of standing two hours in boiling water.	[175]
	Poly (lactic-co-glycolic acid) (PLGA)	PLGA NPs loaded with calcitriol were proven to be prominent enhancers of calcitriol antineoplastic activity in vitro.	[176]
	Inorganic NPs	Vitamin D-loaded gold NPs proved to greatly enhance osteogenic differentiation in vitro.	[177]
		Stable CaCO3-NP-based Pickering emulsion containing vitamin D3 can be regarded as the ultimate supplements since they combine both calcium and vitamin D3, crucial for its absorption.	[178]
		The encapsulation of vitamin D in nano-graphene oxide NPs seems to be dependent on the presence of TW 80 surfactant.	[179]

## Data Availability

Not applicable.
